# A Comparison of Currently Available and Investigational Fecal Microbiota Transplant Products for Recurrent *Clostridioides difficile* Infection

**DOI:** 10.3390/antibiotics13050436

**Published:** 2024-05-12

**Authors:** Yifan Wang, Aaron Hunt, Larry Danziger, Emily N. Drwiega

**Affiliations:** 1 Department of Pharmacy Practice, University of Illinois at Chicago College of Pharmacy, Chicago, IL 60612, USA; 2 Division of Infectious Diseases, University of Illinois at Chicago College of Medicine, Chicago, IL 60612, USA

**Keywords:** live biotherapeutic products, fecal microbiota transplant, microbiome

## Abstract

*Clostridioides difficile* infection (CDI) is an intestinal infection that causes morbidity and mortality and places significant burden and cost on the healthcare system, especially in recurrent cases. Antibiotic overuse is well recognized as the leading cause of CDI in high-risk patients, and studies have demonstrated that even short-term antibiotic exposure can cause a large and persistent disturbance to human colonic microbiota. The recovery and sustainability of the gut microbiome after dysbiosis have been associated with fewer CDI recurrences. Fecal microbiota transplantation (FMT) refers to the procedure in which human donor stool is processed and transplanted to a patient with CDI. It has been historically used in patients with pseudomembranous colitis even before the discovery of *Clostridioides difficile*. More recent research supports the use of FMT as part of the standard therapy of recurrent CDI. This article will be an in-depth review of five microbiome therapeutic products that are either under investigation or currently commercially available: Rebyota (fecal microbiota, live-jslm, formerly RBX2660), Vowst (fecal microbiota spores, live-brpk, formerly SER109), VE303, CP101, and RBX7455. Included in this review is a comparison of the products’ composition and dosage forms, available safety and efficacy data, and investigational status.

## 1. Introduction

*Clostridioides difficile* (*C. difficile*) is a gram-positive, spore-forming bacterium responsible for causing severe infectious diarrhea largely through the production of exotoxins [1]. Symptoms of *Clostridioides difficile* infection (CDI) range from abdominal pain to fulminant, septic disease, with multiple episodes of watery diarrhea as the cardinal symptom [1]. CDI is considered a major healthcare threat that affects nearly half a million Americans and costs up to an estimated USD 4.8 billion in excessive acute care expenses [2,3,4,5,6].

Recurrent CDI (rCDI), which has been observed to occur in up to 35% of all patients after their first occurrence, is a major complication that causes additional morbidity and burden to the healthcare system [7]. rCDI is defined by the Infectious Disease Society of America (IDSA) and The Society for Healthcare Epidemiology of America (SHEA) as a recurrence within 8 weeks of the resolution of the previous CDI episode, despite successful treatment [8]. Following the resolution of an initial recurrence, as many as 45 to 65% of patients may experience additional subsequent recurrences [9,10].

The 2021 IDSA/SHEA guidelines for the management of CDI recommend fidaxomicin 200 mg twice daily for 10 days, or tapered twice daily for 5 days followed by once every other day for 20 days, as the preferred regimen for rCDI. Oral vancomycin is listed as an alternative agent given as 125 mg 4 times daily for 10 days or in tapered dosage courses [8]. Fidaxomicin has demonstrated a reduction in the rate of first recurrences when compared to vancomycin [11]. However, despite guideline recommendations, clinical evidence is lacking in terms of fidaxomicin’s efficacy in subsequent recurrences [12]. Considering the lack of a well-defined prevention strategy for multiple rCDI, managing this disease remains a challenging clinical issue. The IDSA/SHEA guidelines also suggest fecal microbiota transplantation (FMT) as one of the possible alternative therapies for multiple CDI recurrences after the completion of antibiotic treatment [8]. The American Gastroenterological Association and the British Society of Gastroenterology have both published more specific guidelines on FMT with similar recommendations [13,14].

The human gut microbiome is a complex ecosystem of microorganisms within our gastrointestinal (GI) tract, and it has been well described that CDI and its recurrence are closely associated with disruption to the diversity of this microbiome [15,16,17,18,19,20,21]. FMT is a procedure that delivers healthy human donor stool into the intestinal tract of a patient in order to alter the patient’s GI tract microbial composition to a normal (healthy) state. FMT has been studied as a treatment and prevention therapy for multiple rCDI [22]. Before reviewing the recent developments in FMT as treatment and prevention for rCDI, it is important to fully discuss this association between the gut microbiome and CDI. 

## 2. The Human Gut Microbiome and CDI

It is generally believed that a healthy gut environment is dependent on the diversity and density of its microbiome. The term commensal gut microbiome refers to the trillions of microbes, which consist of more than 1000 different species, that normally colonize the human GI tract [23,24,25]. The gut microbiome composition can also be highly variable between individuals [26]. Factors such as diet, geographical location, weight, and exercise level can have a dramatic impact on the microbiome composition [26]. Although the human gut is estimated to host over 100 trillion bacterial cells, only six phyla constitute the majority of the gut microbiome, with *Firmicutes* and *Bacteroidetes* as the two dominant phyla present in the gut of most healthy adults, followed by *Actinobacteria* and *Proteobacteria* [24]. The *Firmicutes* phylum is a large phylum that includes various enteric genera such as *Lactobacillus*, *Enterococcus,* and *Clostridium.* The *Bacteroidetes* phylum also includes the common enteric genera *Bacteroides* and *Prevotella* [25,27]. Besides these microorganisms, certain metabolites may also contribute significantly to the overall health of the microbiome. Bile acids and short-chain fatty acids (SCFAs) are examples of such compounds that are closely involved in maintenance of intestinal integrity, prevention of gut disorders, and modulation of the growth of certain bacterial species within the microbiome [28].

As previously stated, there are certain phyla within the GI tract that are more abundant, and therefore assume more responsibility for maintaining the overall health and balance of the microbiome [28,29,30]. This diversity and balance of the gut microbiome is known to protect the host against pathogenic organisms such as *C. difficile* [27,28,31]. Both *Bacteroidetes* and *Firmicutes* are considered important phyla due to the broad metabolic and physiologic effects they exert [27,28,30]. For example, bacteria species within the *Bacteroidetes* phylum have the ability to synthesize certain lipids that reduce natural killer T cell proliferation, thereby reducing inflammation from immune response [28].

Changes or disruption to the gut microbiome, referred to as dysbiosis, are closely associated with certain diseases, such as type 2 diabetes, inflammatory bowel diseases, and CDI [15,16,17,18]. In human studies, participants with CDI experience a considerable decrease in the amount of *Bacteroides*, *Prevotella*, *Actinobacteria*, and *Bifidobacterial*, while numbers of *Proteobacteria*, *Lactobacillus* and *Clostridium* increase [19,31]. Studies have also identified specific species and byproducts that may provide resistance against *C. difficile* pathogenesis within the GI tract [30]. *Bifidobacterium* species have been shown to decrease the growth and toxicity of *C. difficile* in the presence of SCFAs in vitro [20,32]. Studies have shown that several species of *Bacteroides* not only inhibit the adherence and growth of *C. difficile* within the murine GI tract, but also promotes restoration of commensal microbiome composition [33,34].

The use of broad-spectrum antimicrobials has been documented to be a major risk factor for gut dysbiosis, and consequently CDI, due to the changes in gut microbiome composition after antibiotic exposure [35,36,37]. It has also been demonstrated that even short-term exposure to antibiotics—as few as 7 days—may cause years of long-lasting impact on gut microbiome [38]. Due to the alteration of the commensal microbiome after exposure to antibiotics, *C difficile* spores are given the opportunity to germinate and proliferate in the gut [38]. The most notorious of the antibiotics include fluoroquinolones, clindamycin, and broad-spectrum beta-lactams, which may lead to disproportional changes in the abundance of *Firmicutes* and *Bacteroidetes* [15]. Studies have reported a consistent reduction in both *Bacteroidetes* and *Firmicutes* among patients suffering from rCDI, and *C. difficile* has even become the dominant species in some patients [21,39,40].

Another known consequence of antibiotic exposure to the gut microbiome is the substantial decrease in secondary bile acids, whose presence has been shown to attenuate the virulence and toxigenicity of *C. difficile* [41,42,43,44,45]. Secondary bile acids are converted from primary bile acids by commensal members of the gut microbiome [43]. In vitro studies have demonstrated that certain types of secondary bile acids can inhibit the growth of *C. difficile* spores [46]. It has been shown that antibiotics alter and diminish these commensal organisms, which ultimately leads to a reduction in secondary bile acids in the gut [43]. In patients with initial and rCDI, secondary bile acids have been shown to be significantly reduced compared to before the onset of CDI [46]. The recognition of these processes, as well as the specific organisms and components within the gut microbiome that potentiate gut health, forms the basis for therapies such as FMT [22].

## 3. History of Fecal Microbiota Transplant in Clinical Practice

As previously stated, FMT is a procedure that transfers the stool of a healthy donor to a recipient to restore the commensal gut microbiome with the aim of curing a disease. FMT has ancient roots in traditional medicine practices, but the standardized medical use of FMT only began in the 20th century [47]. One of the first documented case studies of FMT was published in 1958, when Eiseman et al. reported on the successful treatment of four patients with pseudomembranous enterocolitis using a fecal enema [48]. Researchers later discovered the causative relationship between *C. difficile* and pseudomembranous enterocolitis in 1978 [49] A few decades later, clinicians began to explore the application of FMT for the treatment of CDI [50]. The rationale behind this approach is that pathogenic organisms, such as *C. difficile*, are less likely to proliferate once the gut microbiome is restored to a healthy and balanced state [51].

Studies have shown that FMT alters the recipient’s gut microbiome post-transplantation, and that the bacterial contents of the recipient’s stool resemble that of the healthy donor, specifically in the abundance of *Bacteroidetes* [52,53] This persistent alteration is often referred to as stable engraftment and can last for more than 30 days. Researchers demonstrated that, after FMT in three recipients with rCDI, the recipients’ stool bacteria was dominated by *Bacteroidetes* and *Firmicutes* in quantities similar to the healthy donor, while the number of *Proteobacteria* decreased compared to the recipients’ baseline [54].

FMT has been established as a safe and effective procedure for the prevention of rCDI [51]. Fecal samples are usually diluted with sterile liquids and administered soon after preparation. These are typically given rectally as an enema during colonoscopy, or through nasogastric or nasoduodenal tubes [55,56,57,58]. Several published studies have shown sustained clinical response in the prevention of rCDI in patients receiving various preparations of FMT when compared to standard antibiotic regimens [59,60,61,62,63]. For example, Hvas et al. compared FMT with a short course of vancomycin to either a standard course of fidaxomicin or vancomycin in patients with rCDI, and found that FMT combined with vancomycin was superior to fidaxomicin or vancomycin alone in terms of clinical and microbiological resolution [63].

Studies have also compared these various preparations and delivery models of FMT [64,65]. A Canadian study compared fresh to frozen-and-thawed FMT products, and found that frozen FMT was noninferior to fresh FMT in terms of clinical resolution of CDI diarrhea without relapse at 13 weeks, with no difference in adverse events (AEs) [64]. A meta-analysis did demonstrate that the freshly prepared FMT had an overall higher trend of success, but direct comparison showed no statistical difference between fresh, frozen, and lyophilized FMT products [65]. A group of practitioners in the United Kingdom also performed an economic evaluation of FMT and found that FMT was more cost-effective than antimicrobial therapy; delivery through a nasogastric tube was preferred over other routes of administration [66]. 

In the early 2010s, medical centers were performing FMT procedures by procuring donor stool from family members or close relatives of patients, and independent institutional protocols were established for donor screening, specimen preparation, and administration of FMT [58,67,68]. Because of these unregulated and unstandardized practices, the FDA classified FMT as an investigational drug in 2012 [69]. At that time, an Investigational New Drug (IND) application was required for each use, which often had a prolonged turn-around time of months to years [69].

In 2013, during a public meeting, physicians expressed concerns of delays in treatment due to IND requirements, and the FDA began allowing the use of FMT for CDI treatment without IND submission [69,70]. An industry guidance document was also released the same year, as well as multiple other practical guidance documents regarding FMT for rCDI for practitioners [70,71,72]. In 2014, the enforcement discretion policy was updated to include that stool must be obtained from a known donor. In order to avoid transmission of pathogenic organisms, the FDA also required that the stool be screened prior to use [70].

Unfortunately, in June of 2019, the US FDA released a safety alert that described two immunocompromised adults that developed invasive extended-spectrum beta-lactamase (ESBL)-producing *Escherichia coli* infections following FMT. FMT in these two patients was prepared with stool obtained from a single donor, which was later tested and found to be positive for the infective *E. coli* strain in the two patient cases [73]. As a result, additional donor screening questions and stool testing for resistant organisms were required moving forward for the use of FMT [74].

## 4. Live Biotherapeutic Products

In recent years, due to the aforementioned concerns with FMT, pharmaceutical companies have sought to develop standardized FMT products to reduce variability, ensure safety, and improve scalability. Live biotherapeutic products (LBPs) are defined by the FDA as: “(1) contains live organisms, such as bacteria; (2) is applicable to the prevention, treatment, or cure of a disease or condition of human beings; and (3) is not a vaccine [75]”. LBPs differ from conventional FMT due to its recognition by the FDA as a class of biological drug products [75]. It is important to note that FMT is currently recognized by the FDA as an investigational medical procedure instead of a drug product [76]. LBPs must be manufactured under good manufacturing practice methods, and like medications, must undergo rigorous clinical trials before they can be FDA approved [75]. Healthy stool donors are also screened more consistently for possible pathogens, as required by the FDA [75,77].

Clinicians and pharmaceutical companies have been seeking the opportunity to develop more marketable and standardized microbiome-based products. At this time, two LBP products have been approved by the FDA for prevention of rCDI with more in the pipeline. This article will be an in-depth review of products that are either under investigation or currently commercially available: Rebyota (fecal microbiota, live-jslm, formerly RBX2660), Vowst (fecal microbiota spores, live-brpk, formerly SER109), VE303, CP101, and RBX7455. Included in this review is a comparison of the products’ compositions, dosage forms, available safety and efficacy data, and investigational status. Key information is summarized in Table 1 and Table 2. 

### 4.1. Rebyota (RBX 2660)

Rebyota™ (fecal microbiota, live-jslm; previously RBX 2660) was the first FDA-approved LBP indicated for preventing rCDI in patients with one or more recurrences following antibiotic treatment. This product was developed by Rebiotix, a Ferring company (Roseville, MN, USA). Rebyota is an enema comprised of fecal microbes suspended in a solution of polyethylene glycol 3350 and 0.9% sodium chloride [78]. One dose contains a minimum of 1 × 10^5^ CFU/mL Bacteroides and between 1 × 10^8^ and 5 × 10^10^ CFU/mL of total fecal microbes. The product should be stored frozen between −60 °C and −90 °C and moved to a refrigerator within 5 days prior to use. Stool is collected from qualified donors who undergo routine health monitoring, and each stool donation is screened for multi-drug-resistant organisms and enteric pathogens [84]. Following an antibiotic treatment course for rCDI and a 24–72-h washout period, Rebyota is given as a 150 mL rectal suspension for the prevention of CDI recurrence. Rebyota is administered by a healthcare provider via gravity flow in under 15 min and does not require bowel preparation products in advance [93].

The clinical evidence supporting Rebyota is primarily derived from four published clinical trials referred to as the “PUNCH” studies [84,85,86,87]. The standard definition for efficacy in all of the PUNCH trials was the prevention of IDSA/SHEA-defined rCDI at 8 weeks following infusion. PUNCH-CD was a single-arm, open-label trial assessing AEs within 6 months of Rebyota administration as the primary outcome [84]. This trial enrolled participants with at least three CDI episodes or at least two severe CDI episodes, defined as requiring hospitalization. All participants required a positive stool test for *C. difficile* and must have clinically responded to an antibiotic treatment for rCDI. Participants were given Rebyota after a vancomycin lead-in with an optional second dose of Rebyota upon symptomatic recurrence. After completing 6 months of follow-up, 31 of 34 subjects enrolled were eligible for outcome assessment. Of the three participants excluded from assessment, two withdrew from the study and one was an unrelated death. AEs were reported among 28/31 patients, with gastrointestinal symptoms comprising the majority reported (107/188). Fifty-eight and a half percent (110/188) of all reported AEs were determined to be possibly related to Rebyota. No serious AEs were attributed to the study drug. Lack of recurrence was reported in 51.6% (n = 16/31) of participants after the first dose and 78.6% (n = 11/14) of participants receiving two doses, for an overall success rate of 87.1% (27/31) per the study’s authors.

The PUNCH-Open Label study compared the efficacy of two unblinded doses of Rebyota following CDI antibiotic therapy to a historical control with only antibiotics as the primary outcome [85]. The control arm consisted of past patients treated for CDI at the study sites and having at least 8 weeks of documented follow-up. The enrollment criteria matched the PUNCH-CD trial; however, fidaxomicin lead-in therapy was also permitted. The study enrolled 149 patients to receive Rebyota and 104 patients in the historical control arm. The Rebyota arm had 66% of participants enrolled using PCR stool testing, a mean of 3.9 (2–13) total CDI episodes, and 81% of patients treated with vancomycin lead-in therapy. The control arm had 83% of patients enrolled using PCR stool testing, a mean of 2.9 (3–5) total CDI episodes, and 63% of patients treated with vancomycin. The efficacy was 79% (n = 112/142) in the treatment arm versus 31% (n = 23/75) in the historical control arm. A sustained clinical response was demonstrated through 24 months in 90% of patients treated with Rebyota. AEs possibly related to Rebyota, most commonly diarrhea and abdominal pain, were reported in 21% of participants. Nine serious AEs deemed to be possibly related to the study drug occurred in two participants. Both patients experienced severe CDI, with one having ileus, atrial fibrillation, and systemic symptoms, resulting in death. The safety outcomes could not be statistically compared between groups due to incomplete records in the control arm.

The PUNCH-CD2 study was a randomized, double-blind trial assessing the 1- and 2-dose efficacy of Rebyota against a placebo [86]. Three study arms were organized, each receiving doses 7 days apart: group A (2 doses of Rebyota), group B (2 doses of placebo), and group C (1 dose Rebyota, 1 dose placebo). The enrollment criteria matched PUNCH Open Label. A total of 127 patients were enrolled, with 81% diagnosed using PCR (103/127) stool testing, a median of four CDI episodes in groups A and C, and a median of three episodes in group B. Vancomycin was given as a lead-in therapy for 93% of group A (38/41), 91% of group B (40/44), and 88% of group C (37/42). The primary outcome of the difference in efficacy between groups A and B in the intention-to-treat population was not significant (55.6% vs. 43.2%, *p* = 0.2). The only significant difference noted was group C, exhibiting higher efficacy than group B in the per-protocol analysis (87.5% vs. 58.1%, *p* = 0.017) [86]. Treatment-related AEs were similar in frequency across the study arms during the treatment phase (A: 59.1%, B: 61%, C: 72.1%), with three possibly-related serious AEs reported in group A, comprising acute myeloid leukemia, severe abdominal cramping, and constipation requiring hospitalization [86,94]. The trial established 24-month safety data, with no serious AEs occurring during follow-up beyond 8 weeks [86].

The PUNCH-CD3 trial was a phase 3, double-blind, placebo-controlled study that compared the efficacy of a single dose of Rebyota against a placebo, both following antibiotic courses for rCDI [87]. Subjects were monitored for 8 weeks for recurrence and 6 months for safety. Patients with two or more episodes of CDI were enrolled. Difficulty with recruitment necessitated a data-borrowing approach, as recommended by the FDA. The similarity of the PUNCH-2 and PUNCH-3 trial designs allowed for pooling of outcome and safety data. The total population of 267 was divided 2:1, with 180 receiving Rebyota and 87 receiving placebos. Vancomycin lead-in was used in 88% of patients (235/267), 73% had PCR diagnosis, and 63.7% had 2–3 CDI episodes at the time of blinding (170/267). The primary outcome was evaluated by hierarchal Bayesian analysis and resulted in an estimated 12.3% difference in efficacy rates between Rebyota and the placebo (70.4% vs. 58.1%) [87]. The posterior probability of superiority was 0.986, which exceeded the FDA-specified cutoff of 0.975 [87]. A sustained clinical response through 6 months was demonstrated in 90% of patients receiving Rebyota. AEs were reported in 55.6% of the treatment arm and 44.8% of patients in the placebo arm [87]. The disparity was primarily due to mild AEs. Additionally, nine patients experienced serious AEs, but all were deemed unrelated to Rebyota [87].

The enrolled populations across all PUNCH studies were similar and entirely from North America. Participants were approximately 90% white, two-thirds female, and had a mean age of 65 years. Over 90% of all patients had received vancomycin lead-in therapy prior to Rebyota infusion. Rebyota consistently outperformed antibiotic-only treatment regimens for the prevention of rCDI and consistently showed above a 90% sustained clinical response. AEs were similar between the treatment and placebo arms across all studies (69.7% vs. 60.2%), and no pathogen-traced infections were attributed to Rebyota [79]. The efficacy and safety findings were sufficient for the FDA to approve Rebyota as the first LBP on 30 November 2022 [95]. 

### 4.2. Vowst (SER-109)

The second FDA-approved LBP for the prevention of rCDI, and first oral LBP, is Vowst (fecal microbiota spores, live brpk, formerly SER-109), developed by Seres Therapeutics (Cambridge, MA, USA). Vowst should be initiated 2 to 4 days after completing antibiotic treatment for CDI. A one-time, 300 mL oral dose of magnesium citrate should be taken the night before starting Vowst to remove residual antibiotics from the bowel. Vowst is supplied as oral capsules containing 1 × 10^6^ to 3 × 10^7^ CFU of *Firmicute* spores each [7]. The labeled dosing is four capsules taken together prior to the first meal of the day for 3 consecutive days [80]. The *Firmicutes* spores are derived from qualified donors who undergo routine health screening and donor stool testing for pathogens before purification and encapsulation [88,96]. Vowst can be stored between 2 °C and 25 °C for up to 36 months. 

Four trials have provided clinical data supporting the use of Vowst [88,89,90,91]. Standard preliminary treatment was required for patients enrolled in all trials and included any CDI antibiotic course followed by completion of bowel preparation. The initial phase 1 trial published by Khanna et al. was an open-label, two-arm study assessing high- and low-dose Vowst, with the 8-week prevention of rCDI as the primary outcome [88]. Enrolled adults needed to have undergone at least two assay-confirmed CDI recurrences in the preceding 12 months. Polyethylene glycol was used for bowel preparation at two sites; however, all subsequent trials used magnesium citrate only. Participants received a single high-dose (mean 1.7 × 10^9^ spores) or low-dose (1.1 × 10^8^ spores) treatment, and any participant whose diarrhea recurred within the 8-week follow-up period was eligible for a repeat dose. The median for both arms was three recurrences prior to enrollment. Both arms of 15 patients had 86.7% meet the primary endpoint (13/15) after one dose. Of the four patients that did not meet the primary endpoint, three did not require retreatment despite a protocol-defined episode of CDI diarrhea (their diarrhea was deemed not to be caused by *C. difficile* by investigators). The remaining one patient dropped out of the study. Including these three participants, the authors reported an 8-week clinical resolution of 96.7%. Clinical response was maintained in 87% (20/23) of patients who completed a 24-week safety follow-up. Half of the patients experienced mild or moderate AEs that were related to the study drug. None of the eight total serious or severe AEs reported were deemed related to the study drug.

The next study by McGovern et al. was a randomized, double-blind trial evaluating 8-week recurrence prevention as the primary outcome [89]. At least two assay-confirmed recurrences of CDI in the preceding 9 months were required for enrollment [89,97]. Eighty-nine patients were randomized 2:1 to receive four capsules of SER-109 (1. × 10^8^ spores) or placebo after standard preliminary treatment. The SER-109 arm had 35.6% of patients with a history of 3 prior recurrences as compared to 16.7% of the placebo arm. Approximately 20% of patients in each arm received a fidaxomicin antibiotic lead-in, and 80% used PCR stool testing for enrollment. No significant difference in CDI recurrence was observed between Vowst and placebo over 8 weeks (44.1% vs. 53.3%, RR 1.2 [0.8–1.9]). The lack of difference in outcomes between these two groups was thought to be due to the combination of the use of PCR testing for diagnosis in most participants and an underdosing of Vowst. However, subgroup analysis demonstrated significantly lower recurrence for participants aged 65 years or older receiving Vowst (45.2% vs. 80%, RR 1.8 [1.1–2.8]). In patients who received Vowst, 18.3% experienced an AE possibly related to the study drug. The overall incidence of AEs (76.7% vs. 69%) and that of gastrointestinal AEs (55% vs. 44.8%) were comparable between Vowst and the placebo. Serious AEs occurred in 15% of the SER-190 arm and 10.3% of the placebo arm, none of which were deemed related to study interventions. 

ECOSPOR III was a randomized, double-blind trial of similar design to the previous trial [90]. Differences include an intervention dose of four capsules (3 × 10^7^ spores) given over 3 consecutive days and an enzyme immunoassay (EIA) toxin assay requirement for enrollment. Stratification of participants by age (> or <65 years) and antibiotic preliminary treatment (vancomycin or fidaxomicin) was performed. Other enrollment criteria and the primary efficacy outcome were identical to the Khanna S. et al. trial. The number of women enrolled (67% vs. 53%) and history of three or more recurrences (44% vs. 34%) were greater in the SER-109 arm. Fidaxomicin lead-in therapy comprised roughly a quarter of each arm. CDI recurrence was observed in 12% (11/89) of the Vowst arm compared to 40% (37/93) of the placebo arm at 8 weeks, demonstrating superiority (RR 0.32 [0.18–0.58], *p* < 0.001). Secondary analysis confirmed superiority across all stratified populations. The sustained response at 24 weeks was 88% (78/89) for patients treated with Vowst. Seventy-five percent (36/48) of overall recurrences occurred within 2 weeks of intervention. No serious AEs were related to Vowst. AEs possibly related to intervention were similar between arms, and the most commonly reported AEs among patients in both arms were gastrointestinal symptoms such as flatulence and abdominal distension (88% SER109 vs. 87% placebo) [90].

ECOSPOR IV was a phase 3, open-label trial assessing 24-week safety as the primary endpoint. Individuals were enrolled into one of two cohorts [91]. Participants from the ECOSPOR III trial who had rCDI diagnosed via toxin EIA were enrolled in cohort one [91]. Additionally, new adults with at least one CDI recurrence and any positive assay were enrolled into a second cohort. All patients received identical dosing to ECOSPOR III. A total of 263 patients were enrolled, 29 in the first cohort and 234 in the second. Most patients (71%) had two or more CDI recurrences at baseline. Overall, 53.6% (141/263) of patients reported AEs, most commonly diarrhea, flatulence, and nausea. One patient experienced a hypersensitivity reaction to Vowst that was managed with antihistamines and steroids. No other serious AEs or deaths were determined to be related to Vowst by investigators. CDI recurrence over 8 weeks was 8.7% (23/263): 4 patients from cohort 1 and 19 patients from cohort 2. The sustained clinical response at 24 weeks was 86.3% (227/263). Of the 36 patients classified as having recurrent infection through week 24, only 22 had stool assay confirmation, with the remainder being assumed to be treatment failures due to loss of follow-up, early termination, missing components, or death [91].

Across these four Vowst trials, the enrolled participants were two-thirds female, predominantly white, and had a mean age of 65 years. All participants were enrolled from North America. Vancomycin was used for standard preliminary treatment in 70–80% of patients. The treatment-related AEs that occurred were mostly mild to moderate and gastrointestinal in nature. The only serious AE related to the study drug was a hypersensitivity reaction in one patient. Treatment with Vowst resulted in recurrence rates and sustained clinical responses comparable to Rebyota and superior to antibiotic monotherapy when dosed at four capsules daily for 3 consecutive days. These efficacy and safety findings led to FDA approval of Vowst as the first orally administered LBP for the prevention of rCDI on 26 April 2023 [98].

### 4.3. VE303

VE303 (Vedanta Biosciences, Cambridge, MA, USA), is an investigational LBP containing a total of 8 × 10^8^ CFUs from 8 distinct *Clostridial* species, and is administered orally as capsules [99,100]. VE303 is unique in that it does not rely on donor stool, but grows eight different strains of Clostridial bacteria (commensals found in the gut) from bacterial cell banks, thus potentially improving consistency and standardization. In pre-clinical studies, VE303 was shown to increase survival rates in murine models and to significantly inhibit the growth of *C. difficile* in in vitro competition assays [101].

An initial-phase 1a/1b dose-escalating trial was completed in 39 healthy adult volunteers in 2019 [81,101]. Participants received between 1.6 × 10^9^ and 1.1 × 10^11^ CFUs of VE303 in various ascending doses, with or without a preceding course of oral vancomycin as pre-treatment [101]. The study found that VE303 strains were more consistently detected after dosing for multiple days. No serious or severe AEs were reported [101]. Additionally, the authors noted that the administration of VE303 following vancomycin was related to an increase in metabolites and microbial taxa associated with gut colonization resistance. The most common AEs included abdominal distention, diarrhea, nausea, headache, and increased lipase. VE303 was also associated with faster recovery of SCFAs, secondary bile acids, and gut microbiota compared to antibiotics [101]. The authors concluded that the study demonstrated sufficient data to proceed with additional investigational trials. 

Vedanta Biosciences sponsored a phase 2, double-blind clinical trial to investigate the safety and efficacy of VE303 for the prevention of rCDI in individuals with a prior CDI history or following the first occurrence of CDI with study-defined high risk for recurrence [82,92]. In this trial, CONSORTIUM, participants completed a standard course of treatment antibiotics for CDI, and then were randomized to either 14 days of oral VE303 at two different doses or placebo. Seventy-nine adult participants were included: 30 in the VE303 high-dose group (10 capsules daily), 27 in the low-dose group (2 capsules daily), and 22 in the placebo group. The median age across all participants was 62.1 years, and the majority were female (70.9%). The majority of the participants was Caucasian (96.2%). Thirteen participants enrolled had only one prior episode of CDI. Of those who had a history of recurrence, the median number of prior CDI episodes at baseline was three in each group. The primary outcome of rCDI at or before week 8 occurred in 4/29 (13.8%) participants in the high-dose VE303 group, 10/27 (37.0%) in the low-dose VE303 group, and 10/22 (45.5%) in the placebo group. Serious AEs were rare, and the most common AE in all three groups was diarrhea (high dose: 72.41%, low dose: 85.19%, placebo: 86.36%).

Given the reduction in the odds of recurrence demonstrated in CONSORTIUM, a phase 3 trial of VE303 is planned for 2024 and represents the most advanced clinical trial of a bacterial consortium LBP for rCDI [90,92]. VE303 is also currently being explored for the treatment of hepatic encephalopathy (NCT04899115) [102]. Vendanta has stated that VE303 has the potential to become a first-in-class therapeutic based on a defined bacterial consortium, and was granted Orphan Drug Designation in 2017 and Fast Track Designation in 2023 by the FDA for the prevention of rCDI [103].

### 4.4. RBX7455

RBX7455 by Rebiotix (Roseville, MN, USA) is a standardized, lyophilized LBP under investigation for oral administration in individuals with rCDI [83]. This product is directly prepared from RBX2660 in 8 × 10^8^~2 × 10^9^ CFU of fecal microbes. After processing, the product is doubly encapsulated and formulated to open in post-gastric pH conditions [83]. RBX7455 is unique in that it is not frozen and is stored initially at 2 °C to 8 °C until it is dispensed to the patient, where it can then be stored at room temperature for up to 12 months [83]. 

The results from a phase 1 study of RBX7455 for rCDI were published in 2021 [83]. This dose-ranging, open-label trial was designed to assess the safety and efficacy of RBX7455, and included adult patients with at least one recurrence. Participants initiated one of three dosing strategies between 24 and 48 h after completion of treatment antibiotics (Group 1: four capsules twice daily for 4 days, Group 2: four capsules twice daily for 2 days, or Group 3: two capsules twice daily for 2 days). The minimum bacteria CFU per capsule is proprietary. Thirty participants received RBX7455, 10 in each dosing group. The median ages in each group were 67.5, 55, and 63.2 years, respectively, and the number range of prior CDI episodes was 2 to 4. All the participants were of white ethnicity. Three total participants had recurrences at 8 weeks: one in Group 1 (10% recurrence rate) and two in Group 2 (20% recurrence rate), and Group 3 had no reported recurrences. Additionally, no recurrences were reported between 8 weeks and 6 months. Gastrointestinal treatment-emergent AEs were most commonly observed, which included transient diarrhea (10), constipation (5), and abdominal discomfort (3). Two patients experienced serious treatment-emergent AEs, neither of which were attributed to RBX7455 [83]. The study also showed that the microbiome composition in the study participants increased in abundance of Bacteroidia and nonpathogenic Clostridia post-treatment. This shift was also observed with RBX2660 [83].

In addition to rCDI, RBX7455 is also being studied for its immunomodulatory effects in participants with breast cancer and hepatic encephalopathy, as well as in pediatric participants with Crohn’s [104,105,106,107]. Rebiotix is on track to have RBX7455 approved as an oral alternative to Rebyota.

### 4.5. CP101

CP101 (Finch Therapeutics Group, Inc.) is an investigational, single-dose, oral-capsule, donor-derived microbiome product. No information is known currently about the storage or handling requirements of CP101. A phase 2 clinical trial (PRISM3) was conducted to evaluate the safety and efficacy of a single dose of CP101 as compared to placebo [108,109]. A total of 198 participants were included in a modified intention-to-treat (mITT) population of the study, with 102 in the CP101 arm and 96 in the placebo arm. The included participants had a median age of 66.2 years, were predominately female (134/198, 67.7%), and were similar between groups [108]. This study included participants experiencing their first CDI recurrence, but 62.7% in the CP101 arm and 69.8% in the placebo had had three or more prior CDI episodes in the previous 12 months [108]. In the mITT group, 74.5% of participants in the CP101 group did not recur, as compared to 61.5% (*p* < 0.05) in the placebo group through week 8, resulting in a 33.8% relative risk reduction in CDI recurrence [109]. There were no serious treatment-related AEs in the CP101 group [109]. The most common AEs in the CP101 group were diarrhea (54.81%), abdominal pain (34.62%), and defecation urgency (32.69%). In an open-label extension study (PRISM-EXT), all participants with confirmed CDI recurrence were eligible to receive CP101 [110]. A total of 132 participants were analyzed; 50 participants had previously participated in the PRISM3 trial, and 20 out of these 50 participants were treated with CP101. Of all the included PRISM-EXT participants, 80.3% (106/132) had an absence of recurrence through 8 weeks. 

PRISM4 was a phase 3, randomized, double-blind trial in which participants were enrolled 2:1 to CP101 or placebo, with a primary endpoint of CDI recurrence and safety through 8 weeks [108]. In March of 2022, the FDA put a clinical hold on the PRISM4 trial in order to understand the screening protocols for SARS-CoV-2 which the company was using for the donor stool. The FDA removed the clinical hold on CP101 in April of 2022. Unfortunately, PRISM4 was terminated in January 2023 following a press release from Finch Therapeutics Group, Inc. They cited multiple reasons for discontinuing PRISM4, such as the need to “secure additional capital or partnerships to help fund the CP101 program through important milestones, slower than anticipated enrollment in the PRISM4 trial, the harmful impact of ongoing unauthorized use of the Company’s intellectual property, and broader sector trends.” [108,111]. No update on the investigational status of CP101 has been made since early 2023.

## 5. Discussion

The FDA approval of two new LBPs represents an exciting advancement towards improving the management of rCDI. Compared to traditional FMT, these products are better regulated and more standardized in manufacturing and preparation. This standardization, accompanied by the rigorous FDA approval process, offers strong evidence to support the application of LBPs. These products consistently exhibited better efficacy compared to placebo, with evidence of sustained clinical responses. The safety profiles were also encouraging, with no severe AEs nor any pathogen-related infections. LBPs may also positively influence quality of life for patients with rCDI and improve associated conditions such as depression [112,113,114]. There is also an added benefit in terms of ordering and administration compared to traditional FMT, which may reduce barriers to patient access.

Some concerns about these products need to be addressed. Currently approved and investigational LBPs are administered differently, and since there are no head-to-head comparisons, there are no data on which route is more efficacious. Additionally, these LBPs are manufactured differently and have completely different microbial compositions; therefore, we do not know the essential or optimal species that help prevent rCDI. As previously mentioned, the gut microbiome is impacted by geography, ethnicity, and diet. There is a concern that, since these studies were primarily conducted in North America with predominantly white participants (92–94%), it is difficult to predict the applicability of these treatments to other populations. Moreover, there are no clinical studies that directly compare whole-stool FMT and modern LBPs, which makes it difficult to conclude which method has better safety and efficacy. There is also a lack of sufficient data regarding long-term effects on the gut microbiome after either FMT or LBP administration. Most importantly, vigilant monitoring and reporting of LBP-related safety data, including surveillance for resistant organisms, remain crucial until we achieve further understanding of the long-term consequences of microbiome alteration. Lastly, participants with fulminant CDI or immunocompromised individuals were excluded from most CDI LBP trials, which makes it difficult to apply LBP findings to these at-risk populations.

The effectiveness of whole-stool FMT products in preventing recurrence has been well established [22,57,58,115]. When examining the data for LBPs, however, it is noteworthy that the efficacy is lower compared to historical reports for FMT therapy. For example, the PUNCH CD2 trial reported a response rate of 62% to 65%, and PUNCH CD3 predicted an efficacy of 70% [86,87]. The efficacy of Vowst at 24 weeks was also reported to range between 80–87% [90,91]. Previous meta-analyses of whole-stool FMT data up until 2023, including clinical trials, case series, and reports, have indicated an efficacy approaching 90% [84,86,87,94,116].

However, this perceived higher success rate compared to LBPs may not be reproducible across different populations. Each FMT has a largely unknown microbial composition due to the variability of donor stool, and is uniquely prepared by different institutions. Several factors may also be causes of these differences in outcomes between LBPs and FMT. Reporting bias in the FMT studies cannot be dismissed, as successful treatment outcomes might have been more likely to be published in historical cases. Another possibility is that, during the manufacturing process of refining whole stool into LBPs, crucial elements from the stool are lost. Different bacterial compositions were chosen for each LBP based on the aforementioned in vitro and in vivo data, but the ideal targets for selection remain unclear. The question persists regarding which organisms or compounds are necessary and preferable for achieving resolution of rCDI.

Given our concern regarding the inability to create a consistent FMT product and since FMT is still considered an investigational treatment by the FDA, we support the use of these FDA-approved LBPs in patients with multiple rCDI. However, the lack of evidence in patients with primary CDI or first recurrence makes it hard to justify early usage of LBPs. We suggest that LBPs should be considered in patients with two or more recurrences if recurrences occur after tapered courses of either vancomycin or fidaxomicin. 

## 6. Conclusions

The refinement of FMT procedures into commercially available LBPs is a significant advance in the management of rCDI. Clinical results thus far have demonstrated safety and efficacy. More data on the use in both general and at-risk populations, delivery methods, and optimal microbial composition are needed. The success of LBPs for rCDI validates the microbiome as a therapeutic target and heralds an exciting era in the development of microbiome-based therapies.

## Figures and Tables

**Table 1:** Comparison of various live biotherapeutic products.

LBP	Manufacturer	Dosage Form	Microbes in CFU Per Dosage Form	Admixture	Storage and Stability
RBX2660 Rebyota [78,79]	Rebiotix^TM^	150 mL of liquid suspension administered rectally	1 × 10^8^~5 × 10^10^ per mL of fecal microbes, including >1 × 10^5^ of *Bacteroides*	Polyethylene glycol 3350 and 0.9% sodium chloride	Ultracold freezer (−60 °C to −90 °C), up to 5 days in refrigerator (2 °C to 8 °C)
SER109 Vowst [80]	Seres^TM^	Oral capsule	1 × 10^6^~3 × 10^7^ of *Firmicutes* spores	Glycerol in 0.9% sodium chloride	Store at 2 °C to 25 °C Do not store in freezer
VE303 [81,82]	Vedanta^TM^	Oral capsule	~1 × 10^8^ of microbes from 8 distinct *Clostridial* species, for a total content of 8 × 10^8^ microbes	Sucrose, histidine, yeast extract, cysteine, metabisulfite, and microcrystalline cellulose	Long term storage at −20 °C
RBX7455 [83]	Rebiotix^TM^	Oral capsule	8 × 10^8^~2 × 10^9^ of fecal microbes prepared from RBX2660	A proprietary formulation of lyoprotectant and cryoprotectant excipients	Store at 2 °C to 8 °C before dispensing, then room temperature at 23 °C to 27 °C

CP101 is not included here due to a lack of data and discontinuation of trials.

**Table 2:** Summary of clinical trials of various live biotherapeutic products.

Trial References	Enrollment Criteria	Assay Used	Clinical Outcome at 8 Weeks
Clinical trials of RBX2660			
PUNCH-CD [84]	At least 3 CDI episodes and completed at least 2 rounds of antibiotics OR at least 2 severe CDI episodes, defined as requiring hospitalization	Stool test and colonoscopic/histopathologic findings of pseudomembranous colitis	Recurrence observed in 15/31 (48.4%) of the 1-dose group; 4/31 (12.9%) of the 2-doses group
PUNCH-Open Label [85]	Matched PUNCH-CD; fidaxomicin lead-in therapy was permitted	Stool test based on study site availability; majority PCR ^1^ and EIA ^2^ for toxin a/b	Recurrence observed in 30/142 (21.1%) of the Rebyota group; 52/75 (69.3%) of the historical control group; *p* < 0.0001
PUNCH-CD 2 [86]	Matched PUNCH Open Label	Stool test based on study site availability	Recurrence observed in 20/45 (43.2%) of the Rebyota + placebo group; 20/45 (43.2%) of the Rebyota group; *p* = 0.243; 25/44 (56.2%) of the placebo group; *p* = 0.201
PUNCH-CD 3 [87]	At least 2 or more episodes of CDI and completed at least 1 round of antibiotics	Stool test of PCR, EIA for toxin a/b, GDH ^3^, or other assays	Recurrence observed in 29.6% of the Rebyota group; 41.9% of the placebo group; posterior probability of superiority was 0.986
Clinical trials of SER09			
Initial phase 2 trial [88]	At least 3 CDI episodes in the preceding 12 months	Stool test based on availability	Recurrence observed in 2/15 (13.3%) of the high-dose group; 2/15 (13.3%) of the low-dose group
ECOSPOR [89]	At least 3 CDI episodes in the preceding 9 months	Stool test of PCR, EIA for toxin a/b, and GDH	Recurrence observed in 44.1% of the Vowst group; 53.3% of the placebo group; no significance achieved
ECOSPOR III [90]	Matched ECOSPOR	Matched ECOSPOR plus EIA for toxin a/b	Recurrence observed in 11/89 (12%) of the Vowst group; 37/93 (40%) of the placebo group; *p* < 0.001
ECOSPOR IV [91]	Cohort 1: matched ECOSPOR III Cohort 2: at least 2 episodes of CDI	Cohort 1: Stool test of EIA for toxin a/b Cohort 2: any positive stool test	Recurrence observed in 4/29 (13.8%) of cohort 1; 19/234 (8.1%) of cohort 2
Clinical trials of VE303			
CONSORTIUM [82,92]	At least 1 CDI episode in the last 6 months AND those with study-defined high risk for recurrence	Stool test of EIA for toxin a/b, CNNA ^4^, PCR, and toxigenic culture	Recurrence observed in 4/29 (13.8%) of the high-dose VE303 group; 10/27 (37.0%) of the low-dose VE303 group; 10/22 (45.5%) of the placebo group
Clinical trials of RBX7455			
Initial phase 1 trial [83]	At least 2 CDI episodes and completed at least 2 rounds of antibiotics	Stool test of EIA for toxin a/b and NAAT ^5^	Recurrence observed in 1/10 (10%) of 4 capsules twice daily for 4 days; 2/10 (80%) of 4 capsules twice daily for 2 days; 0/10 (0%) of 2 capsules twice daily for 2 days

^1^ Polymerase chain reaction; ^2^ enzyme immunoassay; ^3^ glutamate dehydrogenase; ^4^ cell cytotoxicity neutralization assay; ^5^ Nucleic Acid Amplification Test; CP101 is not included here due to lack of data and discontinuation of trials.
